# Supplementary material for: Evolution of antibody titers after two doses of mRNA Pfizer-BioNTech vaccine and effect of the third dose in nursing home residents
Source: PLoS One. 2023 Mar 8;18(3):e0282388. doi: 10.1371/journal.pone.0282388 (PMC9994681; doi:10.1371/journal.pone.0282388)
Supplement: S1 Questionnaire — (PDF) [file pone.0282388.s001.pdf]

ENCUESTA ESTUDIO POSTVACUNACIÓN.

NOMRE Y APELLIDOS:

FECHA DE NACIMIENTO:

1.- Ha pasado la COVID? SI NO

Si la respuesta es afirmativa, indique la fecha de la infección:

Sintomático SI NO

2.- Vacunación

a.- Indique la fecha en la que se le suministró la vacuna:

1º dosis:

2º dosis:

Efectos secundarios (1º dosis – 2ª dosis):

Dolor en la zona de inoculación	SI - SI	NO - NO
Inflamación en la zona de inoculación	SI - SI	NO - NO
Cefalea	SI - SI	NO – NO
Febrícula	SI - SI	NO - NO
Fiebre (>38ºC)	SI - SI	NO - NO
Cansancio	SI - SI	NO - NO
Escalofríos y nauseas	SI - SI	NO - NO
Mareos	SI - SI	NO - NO
Adenopatías	SI - SI	NO - NO

Otras (describir):

Ha tomado paracetamol profiláctico (antes de la vacunación):

SI NO

Ha tomado paracetamol sintomático (primeras 24 horas tras la vacunación):

SI NO

Enfermedades de base/Medicación habitual.
